# Orthodontic and General Dentistry Fear in 8–73-Year-Old Patients at a Large, Urban U.S. Orthodontic Clinic: Self-Reported Point Prevalences and Clinical Implications

**DOI:** 10.3390/healthcare13151775

**Published:** 2025-07-22

**Authors:** Richard E. Heyman, Kelly A. Daly, Charlotte M. Guerrera

**Affiliations:** 1 Family Translational Research Group, New York University, New York, NY 10016, USA; kelly.daly@nyu.edu; 2 College of Dentistry, New York University, New York, NY 10010, USA; cmg674@nyu.edu

**Keywords:** dental fear, orthodontic fear, fear of general dentistry

## Abstract

**Background/Objectives**: Dental fear affects about one in four general dentistry patients in the U.S. and other high-income countries. However, the prevalence of fear in orthodontic practice has received scant attention, with no studies in the U.S. The aim of this study was to investigate the prevalence of orthodontic and general dentistry fear and the relationship between the two among patients at a large, U.S. urban university orthodontic clinic serving a culturally and ethnically diverse population. **Methods**: Patients (*N* = 186) rated their general dentistry and orthodontic fear using a validated single-item scale. **Results**: A substantial proportion of patients experienced clinically significant fear of dentists (22.1% [95% CI 16.31–28.69%]) and orthodontists (17.2% [95% CI 11.61–22.82%]). There was a strong effect size (r = 0.67) between ratings of fear of dentists and orthodontists. Our prevalences were nearly identical to the weighted prevalences in the literature for general dentistry and orthodontic fear (22.90% [95% CI: 20.73–25.22%] and 17.65% [95% CI: 15.09–20.53%], respectively) among orthodontic patients. **Conclusions**: Despite orthodontic procedures being generally less fear-inducing than general dentistry, orthodontists should assume that over one in six patients will be fearful. Further research is needed to create an assessment of the most feared orthodontic stimuli and to broaden the application of evidence-based dental fear treatments. We recommend screening all orthodontic patients using a single, validated question; if patients are fearful, providers should use empathic communication and accommodate patient needs in treatment sessions.

## 1. Introduction

Dental fear poses a significant public health challenge globally, affecting hundreds of millions of people and often leading to adverse oral health outcomes and reduced quality of life across the lifespan [1,2]. In the U.S., 20–25% of adult general dentistry patients present with moderate-to-severe dental fear [3,4,5]. Dental fear often leads to avoidance behaviors [6], which can result in delayed dental procedures, increased need for emergency dental services, and oral health decline—all of which exacerbate fear [7]. Thus, fear and avoidance are mutually reinforcing, leading to what has been labeled the “vicious cycle of dental fear” [8]. Given the functional impairment and oral health complications associated with dental fear, its management has received considerable attention in the literature [9,10]. (Technically, when adaptive responses are to immediate threats, they are labeled as “fear,” and when they are to future threats, “anxiety.” Because the field most frequently labels both as “dental fear,” in this paper, we use that term to comprise both fear and anxiety).

The role of patient fear in orthodontic practice is less well understood [11]. Replication and extension of prevalence research in high-income contexts is especially needed to inform global oral health strategies because only two orthodontic fear prevalence studies are from high-income countries [Canada and Spain], with four others from lower- and lower-middle-income countries on the Indian subcontinent. Replicating prevalence comparisons of both orthodontic and general dentistry fear in orthodontic patients would be informative because specific fears within these contexts appear to differ, with general dentistry patients’ top three fears being painful or uncomfortable procedures, injections, and having an unsympathetic or unkind dentist [12], whereas orthodontic patients’ top fears are extractions and injections [13].

### Prevalence of Orthodontic Dental Fear

As shown in Table 1, rates of general dentistry fear in orthodontic patients were 12.87–38%—weighted prevalence (*N* = 1342) = 22.90%, 95% CI: 20.73%—25.22%) based on one study from Canada [14], one from Spain [15], two from Pakistan [16,17], and one from India [18]. Orthodontic fear among patients in treatment was 8.75% in a study from Nepal [19].

In the only study to compare fear of both settings [14], Canadian orthodontic patients (75% ongoing and 25% new) reported fairly similar levels of fear in both settings (22.8% of general vs. 18.7% of orthodontics). Although substantial overlap exists between general dentistry and orthodontics from patients’ perspectives (e.g., clinic setting, basic exam experiences, similar armamentarium, potential for unpleasant tastes and discomfort), their treatments are fundamentally different. This is evident in both typical visit procedures and the common tools and stimuli to which patients are exposed. For example, the use of needles and injections (the most commonly feared aspect of dental care [20]) is routine during general dentistry procedures but is much less common during orthodontic visits.

Furthermore, patients who undergo orthodontic treatment often seek it for aesthetic or functional improvement or because of a recommendation from a general dentist [21]. Thus, patients’ pursuit of orthodontic care is often desired, even if they understand that some discomfort may be involved. Additionally, patients typically see the orthodontist every 4–8 weeks during treatment, which lasts about two years on average. In contrast, they may only see a general dentist every six months or more. The higher frequency of orthodontic visits gives patients a greater opportunity for exposure to and mastery of the setting (i.e., reduction of catastrophic beliefs and reconsolidation of inhibitory learning [22]), which can lead to decreased fear levels [11]. In contrast, the infrequent nature of general dentistry appointments fails to provide patients with the same opportunity to amass corrective learning experiences [23], thus making it less likely for fear to extinguish on its own.

**Table 1:** Fear of general dentistry and orthodontics in previous studies and the current study.

Authors	Measure[s] Used to Assess Dental Fear	Study Country (World Bank Income Level)	General Dentistry Fear Prevalences	Orthodontic Dental Fear Prevalences
Khokhar et al., 2015 [16]	Dental Anxiety Scale [24]	Pakistan (Lower-middle income)	*N* = 233 None/Mild/Moderate: 87.12% (95% CI: 82.52–90.73%) High: 8.58% (95% CI: 5.56–12.82%) Severe/Extreme: 4.29% (95% CI: 2.29–7.82%) Overall Prevalence: 12.87% (95% CI: 9.30–17.58%)	-
Jain & Pandian, 2016 [18]	Dental Anxiety Scale [24]	India (Lower-middle income)	*N* = 200 None/Mild/Moderate: 77.5% (95% CI: 71.30–82.72%) High: 17% (95% CI: 12.33–22.95%) Severe/Extreme: 5.5% (95% CI: 3.06–9.68%) Overall Prevalence: 22.5% (95% CI: 17.20–28.76%)	-
Roy & Dempster, 2018 [14]	Modified Dental Anxiety Scale [25], Dental Anxiety Scale —Ortho [14]	Canada (High income)	*N* = 675 None/Mild/Moderate: 77.2% (95% CI: 73.91–80.19%) High: 18.7% (95% CI: 15.86–21.90%) Severe/Extreme: 4.1% (95% CI: 2.87–5.82%) Overall Prevalence: 22.8% (95% CI: 19.86–26.06%)	*N* = 675 None/Mild/Moderate: 72.3% (95% CI: 68.86–75.48%) High: 15.1% (95% CI: 12.44–18.23%) Extreme: 3.6% (95% CI: 2.45–5.25%) Overall Prevalence: 18.7% (95% CI: 15.86–21.90%)
Acharya et al., 2021 [19]	Dental Anxiety Scale —Orthodontics [19,24]	Nepal (Low income)	—	*N* = 80 None/Mild: 26.25% (95% CI: 17.65–37.15%) Moderate: 65.0% (95% CI: 53.68–74.92%) High: 7.5% (95% CI: 3.64–14.86%) Severe/Extreme: 1.25% (95% CI: 0.22–6.84%) Overall Prevalence: 8.75% (95% CI: 4.54–16.03%)
Taqi et al., 2023 [17]	Dental Fear Survey [26] (Urdu Translation [17]	Pakistan (Lower-middle income)	*N* = 54 Low: 72.5% (95% CI: 59.20–82.88%) Moderate: 18.5% (95% CI: 10.42–30.68%) High: 0% (95% CI: 0.00–6.64%) Overall Prevalence: 18.5% (95% CI: 10.42–30.68%)	-
Serrat-Lacasta et al., 2025 [15]	Dental Anxiety Index–Short Form [27]	Spain (High income)	*N* = 180 None/Mild = 62% (95% CI: 54.51–69.04%) Moderate = 25% (95% CI: 19.34–31.95%) Extreme = 13% (95% CI: 8.72–19.00%) Overall Prevalence: 38% (95% CI: 31.06–45.45%)	-
Current Study	Modified Gatchel [3,28]	U.S. (High income)	*N* = 186 None-to-mild (0–3): 78.0% (95% CI: 71.74–83.21%) Moderate (4–6): 14.0% (95% CI: 9.69–19.82%) Severe (7–10): 8.1% (95% CI: 4.90–13.06%) Overall Prevalence: 22.1% (95% CI: 16.79–28.61%)	*N* = 186 None-to-mild (0–3): 82.8% (95% CI: 77.0–88.6%) Moderate (4–6): 11.7% (95% CI: 7.0–16.4%) Severe (7–10): 5.4% (95% CI: 2.3–8.5%) Overall Prevalence: 17.2% (95% CI 11.61–22.82%)
Weighted Prevalence (excluding current study)			*N* = 1342 Overall Prevalence: 22.90% (95% CI: 20.73–25.22%)	*N* = 755 Overall Prevalence: 17.65% (95% CI: 15.09–20.53%)
Weighted Prevalence (including current study)			*N* = 1528 Overall Prevalence: 22.80% (95% CI: 20.77–24.97%)	*N* = 941 Overall Prevalence: 17.56% (95% CI: 15.26–20.12%)

Note: 95% confidence intervals (CIs) were calculated using the Wilson approach [29].

Given the paucity of dental fear research in orthodontics, we conducted a study to assess the frequency of orthodontic and general dentistry fear and the relationship between the two among patients in a large, U.S. urban university clinic serving a culturally and ethnically diverse population. No study of this type has ever been conducted in the U.S., providing an opportunity to replicate and extend the findings from the two other studies from high-income countries [14,15] that reported widely divergent results. We hypothesized that fear of general dentistry would be significantly higher than fear of orthodontics. A better understanding of the frequency of fear of orthodontics relative to fear of general dentistry and knowledge of how related the two are can inform dental professionals’ patient care and the multidisciplinary development of disseminable digital versions (i.e., computerized, mobile-app, and telehealth approaches [30,31,32] of evidence-based cognitive-behavioral interventions for dental fear.

## 2. Materials and Methods

### 2.1. Participants

Patients presenting for orthodontic care at the New York University College of Dentistry orthodontics clinic between March 2022 and December 2024 underwent a comprehensive medical and dental history review during their orthodontic intake assessment. The inclusion criteria were (a) a patient presenting for treatment, (b) documented dental fear level assessment (a required question not always completed), (c) for patients 18 years and older, demonstrated competency to provide informed consent, (d) for patients under 18 years, provision of parental or legal guardian consent, (e) sufficient cognitive and language abilities to understand medical/dental history questions. There were no exclusion criteria (such as medical/psychiatric conditions or medication) because the aim of the study was to assess the prevalence of fear, not the etiology. Because this was an archival study, there was no research recruitment process; information was obtained from the electronic health record. The study team had no interaction with the participants and no access to any private health information or other information regarding the participants. Consequently, the New York University institutional review board ruled that—pursuant to the U.S. Department of Health and Human Services [33] Common Rule (45 CFR § 46.104[d][4]—it was exempt from human subjects review.

### 2.2. Measure

Ratings were made using a modified Gatchel [28] single-item scale, which asks the following: ‘On a scale of 0–10, how do you feel about going to the dentist, where 0 is no fear, 4 is moderate fear, and 10 is extreme fear’ [3]. This scale has been shown to have comparable predictive validity as more complex questionnaires [34]. It is face valid and has established convergent [35,36], discriminant [37], concurrent [38], and criterion validity [39], and is not associated with biased socially desirable responding [37]. Single-item dental fear scales exhibit moderate-to-strong convergence with longer scales (such as the ones used in the other four studies) in both adults [40] and children [41].

### 2.3. Procedure

After discussing potential treatment plan options or before an orthodontic treatment appointment, the patients were verbally asked by their orthodontic resident (*N* = 24) to rate their fear of the dentist and their fear of the orthodontist. Child and adolescent patients reported their own fear (i.e., caregivers were not asked to provide fear ratings).

### 2.4. Statistical Analysis

We calculated the confidence intervals for sample sizes to determine a minimum sample size that would balance precision and viability. We stipulated that the minimum would have to fall within the range of the four clinic samples (*N*s = 69, 81, 147, 150) found in a meta-analysis of child/adolescent dental fear studies [42] and above the second lowest *N* (i.e., *N* = 80) of the other studies in orthodontics [19]. With a 95% confidence level and a minimum *N* = 100, the confidence interval of a prevalence of 20% would be 12.67%–29.18%, providing an adequate initial estimate while requiring a feasible number of clinical patients to assess. Basic descriptive statistics, correlations, and between-group significance testing (via *t*-tests) were performed using SPSS Version 28.

We accounted for missing data (*N* = 7 were missing a single variable) using multiple imputation, as deleting cases with missing data can introduce bias and reduce statistical power. Multiple imputation retains all the participants and creates several plausible datasets that account for the uncertainty of the missing information. We generated five complete datasets, analyzed them separately, and then combined the results.

## 3. Results

### 3.1. Participant Demographics

Patient gender identification was 46% male and 54% female, and their ages ranged from 8 to 73 (*M* = 26.12, *SD* = 15.78, Median = 18.50), with 55% being 21 years old or younger. See Table 2 for an age breakdown.

### 3.2. Rates of General Dentistry and Orthodontic Fear

Results of the screening revealed a large segment of the patient sample with clinically significant fear (as indicated by the cut-point of four or above on the modified Gatchel, [28] rating), with 22.1% (95% CI 16.31–28.69%) and 17.2% (95% CI 11.61–22.82%) of patients reporting moderate-to-severe fear of the dentist and orthodontist, respectively. (See Table 1 for the complete breakdown of patient fear classifications.) There was no significant difference between the proportions of individuals reporting moderate-to-severe fear of the dentist and the orthodontist (*z* = 1.312, *p* = 0.19).

General dentistry and orthodontic fear were significantly and strongly positively associated (*r* = 0.67, *p* < 0.001). (See Table 3 for dental and orthodontic fear crosstabulations.) Ten percent of participants rated their fear of the orthodontist (*M*ortho = 5.39, *SD* = 2.52) higher than their fear of the general dentist (*M*dentist = 1.93, *SD* = 1.99). Twenty-three percent rated their fear of the dentist (*M*dentist = 5.29, *SD* = 2.62) higher than their fear of the orthodontist (*M*ortho = 2.41, *SD* = 2.26). Among the remainder of the participants who rated their dental and orthodontic fear equivalently, 3.2% (*n* = 4) endorsed moderate fear of both, and 1.6% endorsed severe fear of both (*n* = 2), with the remaining 95% endorsing none-to-mild dental fear (*n* = 119). Among those with clinically significant (i.e., moderate to severe) dental fear (*n* = 41), 51% (*n* = 21) also had clinically significant orthodontic fear. Among those clinically significant orthodontic fear (*n* = 32), 66% (*n* = 21) reported similar fears of the dentist.

### 3.3. Predictors of Fear

No significant gender differences were found for fear of general dentistry or orthodontics. This was also true for those who met the threshold for clinically significant general dentistry fear and clinically significant orthodontic fear. Similarly, the percentage of females and males with clinically significant levels of general dentistry and orthodontic fear did not differ. Finally, age was not significantly associated with general dentistry or orthodontic fear. This was the case when age was considered continuously, as above, and when differences in general dental fear and orthodontic fear were considered as a function of patients’ developmental stages (i.e., childhood, adolescence, young adulthood, middle age, or older adulthood).

## 4. Discussion

A considerable number of orthodontic patients at our U.S. urban, university-based clinic reported clinically significant (i.e., moderate to severe) fear of the general dentist (22.1%) and orthodontist (17.2%). These rates were nearly identical to those of the only other study to examine fear of both services among Canadian orthodontic patients [14], which reported 22.8% and 18.7%, respectively. They are also nearly identical to the weighted prevalences of the other existing studies: 22.90% (CI: 20.73–25.22%) and 17.65% (CI: 15.09–20.53%), respectively. Although replication is necessary, the closely replicated North American general dentistry and orthodontic fear ratings of orthodontic patients may portend similar findings in other high-income countries, where only one other study, an outlier on prevalence, exists [15].

For orthodontic fear, the prevalences in both the North American studies were considerably higher than that found in the Nepalese study [19] (8.75%), which involved existing orthodontic patients and used a version of the Dental Anxiety Scale (DAS [24]) modified with orthodontic stimuli. In contrast, for general dentistry fear, both the North American studies reported rates (22.1–22.8%) that were nearly identical to the study of general dentistry fear among orthodontic patients in India [18]—which used the DAS [24]—and slightly higher than the extremely small study in Pakistan (18.5%) that used a translated version of the Dental Fear Survey [26]. All were divergent from the DAS study in Pakistan (12.87% [16]) and the Dental Anxiety Index—Short Form [27] study from Spain [15], which were extremely divergent (*z* = −4.52, *p* < 0.0001 and *z* = 4.14, *p* < 0.0001, respectively) from all the other studies, including ours. Although it is not possible to disentangle prevalences from measurement effects, cultural effects, and setting effects (including the country’s income level), those reported by both Khokhar et al. [16] and Serrat-Lacasta et al. [15] are extreme outliers.

The severity of fear of orthodontics and general dentistry was highly related (i.e., a strong effect size between the two). Notably, 10% of the participants rated their fear of the orthodontist higher than their fear of the dentist, whereas 23% reported higher fear levels for the dentist. This variation suggests that individual patients may experience fear differently in these two contexts. Further exploration into the factors contributing to these differences would be valuable for understanding patient experiences and tailoring interventions accordingly [13]. For example, Armfield’s [43] Index of Dental Anxiety and Fear scale comprises both a dental fear module and a 10-item module on the stimuli that general dentistry patients fear; a parallel module of orthodontic fear stimuli would be useful for both researchers and clinicians.

These results also suggest an opportunity for orthodontists to screen for dental fear and, if revealed, discuss it with their patients, assess what they fear will happen, provide corrective experiences [22,44], and help ensure that patients are connected to and receive the general and specialty oral health treatment they need. The unpredictability of care is a hallmark fear for patients in general dentistry [20] and orthodontic [14] settings. Therefore, it is important that both orthodontists and dentists take the time to clearly convey treatment plans and discuss patient reservations and apprehensions to ensure that patients have a reasonable understanding of their treatment [14,19]. The patients in our study were asked about their level of fear during the initial consultation visit at the orthodontics clinic. As such, many of them were unfamiliar with what is involved in orthodontic treatment and may have been referred by a general dentist already knowing potential need for extractions or jaw surgery as part of their treatment, which may have influenced their response to this question.

Regarding demographic factors, we did not find significant differences in fear levels between the male and female patients for fear of either the dentist or the orthodontist, nor an association with age. Some readers may find this surprising, as the literature reports that female patients typically report higher levels of dental fear than males [45], and meta-analyses in children [46] and adults [47] report a significant inverse relationship between age and fear. Nevertheless, age and gender were not associated with general dental or orthodontic fear, replicating Roy and Dempster’s study [14] for both types of fear, and Jain and Pandian [18], Khokar et al. [16], and Serrat-Lacasta et al. [15], who measured only general dental fears in orthodontic patients. Only Acharya and colleagues’ [19] study, which was also an outlier on prevalence, found differences for younger age and females in orthodontic fear. Thus, in orthodontic patients, both general dentistry and orthodontic dental fear appear to be stable across patients’ ages. The fact that our sample had a broader age range than that seen in many orthodontic settings should be viewed as a study strength for examining its invariance across the lifespan. Because orthodontists’ youngest patients are school-age children (typically beginning around age 8–9 when sufficient permanent teeth have erupted), with the heaviest concentration in adolescent and emerging-adult age ranges (e.g., our sample’s median age of 18.5 years), the consistent lack of an age–fear relationship in orthodontic studies may be due to orthodontics’ unique age characteristics.

This study has some limitations. First, the sample size was small and comprised patients from a single university clinic. However, this is typical for clinical studies, and our sample was larger than or comparable to four of the other five studies [16,17,18,19]. Second, the study relied on self-report measures, which are subject to potential biases and limitations. In particular, the fear screening was performed by the orthodontic residents themselves, which could have increased response bias. However, the potential social desirability bias (i.e., downplaying one’s fear of the provider to the provider) would imply that true rates of dental and orthodontic fear are higher than those reported in this study. Because the rate of general dentistry fear in this study is consistent with general dentistry fear in comparable orthodontic settings [14,18]—and those reported in U.S. general dentistry clinical settings [3,4]—the ratings given are likely accurate representations of patients’ general dentistry fear. Third, we did not collect qualitative information to provide more context about the specific nature of the fears being reported; previous studies have found that the most common fears in orthodontic treatment are unpredictability and previous trauma [14], communication and provider relationship factors [14], fear of extractions and injections, and painful procedures [13,14].

### Future Directions

First, as mentioned above, the development of a content-valid assessment [45] of orthodontic fear stimuli—parallel to that for general dentistry in the Index of Dental Anxiety and Fear [43]—is needed.

Second, longitudinal assessment of orthodontic fear (at multiple points across the years-long course of treatment) is needed, particularly one comparing the levels of fear reported at the initial consultation appointment (when fear of the unknown would be greatest) to the levels of fear (a) after the treatment plan has been discussed with the patient, (b) across treatment, and (c) after treatment has been completed. Fear during the treatment planning and early treatment phases may differ depending on the possibility of invasive treatment needed (e.g., extractions requiring injections of local anesthesia, two of the most feared events in dentistry [46,47]). On the other hand, fear levels tend to decrease over time as patients become more accustomed to their orthodontist and orthodontic treatment [11]. Our study is the only one to measure orthodontic fear at intake, as opposed to existing patients [19] or a combination of new and existing patients [14]. Indeed, Roy and Dempster [14] found that cross-sectionally, fear was highest in the patients who had not yet begun orthodontic treatment.

Third, five of the six top fears in general dentistry involve doctor–patient interaction (in declining order: extreme helplessness, extreme embarrassment, dentist not understanding, impolite/rude dentist, and dentist not providing sufficient information [46,47]); these are similar to two of the three fear factors for orthodontics [14] (i.e., patient’s perceptions and relationship with orthodontist or staff). In Table 4, we list orthodontic fears from studies that investigated specific stimuli [13,14,15,48,49]. To derive a maximally powerful but briefer scale, researchers might consider a program of research: (a) conduct exploratory factor analysis to identify underlying fear dimensions [14] and retain the highest loading items from that factor for further development, (b) employ item response theory analyses to retain the most powerfully discriminative items in the briefest scale [50], and (c) use differential item functioning analysis to ensure measurement invariance across demographic groups [51]. Finally, if the content-valid orthodontic stimuli measure identifies similar themes related to patient–team communication (e.g., the “control, trust, and patient–team relationship” construct), studies observing provider–patient communication [52] at different stages of treatment would be informative.

Fourth, dental fear appears to result from at least two pathways: (a) a traumatic event combined with a perception of the lack of control [53] and (b) no personally experienced traumatic dental event [54] but with fears related to painful/uncomfortable, poor dentist–patient interaction, managing physical sensations (e.g., gagging), non-dental traumas (e.g., sexual assault), and hearing about others’ bad experiences [54,55]. Although cultural and economic differences impact both the prevalence of traumatic experiences [55] and the expectations of how professionals and patients should act, patients across disparate cultures all value provider empathy and shared decision-making [56]. More research is needed on how income (personal and societal) and culture impact experiences and doctor–patient communication influence dental fear in both general dentistry and in specialty areas such as orthodontics.

Fifth, we had access to a dataset that included only patients with dental fear responses; thus, we cannot empirically test the possible effect of missingness on our results. However, we have extensive experience with testing missingness on dental fear responses from other clinics’ electronic health records data at our institutions and have never encountered situations in which missingness on dental fear was related to patient age, gender, race/ethnicity, or insurance status. Furthermore, our fear prevalences (a) replicate the other orthodontic/general dentistry prevalence study [14], (b) closely match the weighted average of general dentistry fear in orthodontics settings, and (c) are consistent with the prevalence reported in a large study in the general dentistry clinics of our institution [3]. Thus, these indicators imply that the dental fear of non-included orthodontic patients is missing at random [57]. In other words, the absence of dental fear data for non-included patients would likely be due to orthodontists’ incomplete documentation rather than being systematically related to patient characteristics or their actual fear levels, which would have biased the prevalence estimates.

Finally, mixed-method studies (i.e., combining objective measures and qualitative approaches) could provide a more comprehensive understanding of fear experiences in general-dental and orthodontic settings.

## 5. Conclusions

More than one in six orthodontic patients reported clinically significant fear of the orthodontist, and 22.1% reported similar fears of the dentist. These findings replicate results from the only other comparable study [14] (also conducted in a high-income, North American country) and the weighted prevalences of the extant literature. Although orthodontic procedures are generally less fear-inducing than those in general dentistry, orthodontists should assume that over one in six patients will be fearful. Further research is needed to identify the most feared orthodontic stimuli and to extend evidence-based fear treatment methods. [10]. We recommend screening all patients with a single reliable and valid (yet time-efficient) dental fear question. If patients are fearful, an empathic evocation of patient concerns [58] is indicated, employing motivational interviewing “spirit” [58]—such as partnering (e.g., working with, not on, the patient; viewing the patient as an expert on themselves and their own needs and desires), acceptance (e.g., believing in the absolute worth of the patient, empathizing with concerns), empowerment (i.e., supporting patient autonomy, affirming strengths and efforts)—as well as its basic communication skills (i.e., open-ended questions, affirming, reflecting, summarizing). After establishing a collaborative, trusting relationship, the orthodontist should then compassionately agree to tailor treatment sessions to (a) foster patient comfort and self-efficacy (e.g., stop signals) and (b) disconfirm fear-based cognitions [22,44]. Finally, digital modes of disseminating evidence-based treatments [30,31,32] should be extended to fear in dental specialty settings. Only then can health professionals enhance patient care, improve treatment outcomes, and alleviate the negative impact of fear on oral and systemic health.

## Figures and Tables

**Table 2:** Percentages of patients by developmental category (*N* = 186).

Developmental Category	Ages	*n*	Percent
Children	8–12 years	28	15.1%
Adolescents	13–18 years	65	34.9%
Young Adults	19–39 years	52	28.0%
Middle Age	40–50	20	10.8%
Older Adults	51–73	21	11.3%

**Table 3:** Severity of general dental and orthodontic fear among patients (*N* = 186).

General Dentistry Fear Level (with Modified Gatchel [28] Range)	Orthodontic Fear Level (with Modified Gatchel [28] Range)		
	None-to-Mild (0–3)	Moderate (4–6)	Severe (7–10)
None-to-mild (0–3)	134 (72.0%)	8 (4.3%)	3 (1.6%)
Moderate (4–6)	16 (8.6%)	7 (3.8%)	3 (1.6%)
Severe (7–10)	4 (2.2%)	7 (3.8%)	4 (2.2%)

**Table 4:** Orthodontic fear stimuli noted in the literature.

Control, Trust, and Patient–Team Relationship Issues [14,48,49] Being reprimanded for poor compliance [15] Entry into the operatory unaccompanied [15] Information content, delivery, and sources [49] Intimidation in treatment settings [49] Lack of control during treatment [14,49] Lack of information or knowledge about treatment [14,48,49] Not knowing what to expect or the unknown [14,48,49] Trust in clinical team and patient–clinician rapport [49] Uncomfortable asking questions or fears not taken seriously [14,49] Pain and Procedural Fears [14,48,49] Appliance problems (breaking, poking, losing) [15] Extraction concerns [48] General dental procedures [48] Impressions being taken [14] Injections/needles [13,49] Not achieving desired results [15,49] Pain and discomfort during and after treatment [13,14,15,48] Permanent damage to teeth [15] Recurring problems after treatment [15] Surgery outcomes and impact [49] Surgical risks and procedures [49] Treatment complications and complexity [48] Appearance, Speech, and Social Impact [48,49] Being too old for orthodontic treatment [48] Facial profile and appearance changes [13,15] Social perceptions, judgment, and dating concerns [48,49] Speech changes [15,48] Teasing, bullying, or trivialization by others [49] Unsatisfactory aesthetic outcomes [13,48,49] Practical Life Impact [48,49] Adaptation difficulties [15] Appointment duration and frequency [15] Coping inability [49] Impact on work and life commitments [48,49] Missing important events [49] Not being able to eat anything or changes to eating/chewing [15,48] Oral hygiene challenges [13,15,48] Recovery support [49] Support systems and loneliness [49] Treatment logistics and emergencies [15]

## Data Availability

The data presented in this study are available upon request from the corresponding author (after the signing of a data-use agreement). The data are based on archival health records.

## Abbreviations

The following abbreviations are used in this manuscript:

CI	Confidence Interval
DAS	Dental Anxiety Scale
MDAS	Modified Dental Anxiety Scale
U.S.	United States
